# Roles of the gut microbiota in hepatocellular carcinoma: from the gut dysbiosis to the intratumoral microbiota

**DOI:** 10.1038/s41420-025-02413-z

**Published:** 2025-04-04

**Authors:** Yiqin Wang, Yongqiang Li, Yong Lin, Chuangyu Cao, Dongcheng Chen, Xianguang Huang, Canhua Li, Haoming Xu, Huasheng Lai, Huiting Chen, Yongjian Zhou

**Affiliations:** 1 Department of Gastroenterology and Hepatology, The Second Affiliated Hospital, School of Medicine, South China University of Technology, Guangzhou, China; 2 Department of Gastroenterology and Hepatology, Guangzhou First People’s Hospital, School of Medicine, South China University of Technology, Guangzhou, China; 3 Department of Gastroenterology and Hepatology, Baiyun Hospital of Guangzhou First People’s Hospital (The Second People’s Hospital of Baiyun District), Guangzhou, China

**Keywords:** Cancer microenvironment, Microbiology

## Abstract

Hepatocellular carcinoma (HCC) is closely linked to alterations in the gut microbiota. This dysbiosis is characterized by significant changes in the microbial population, which correlate with the progression of HCC. Gut dysbiosis ultimately promotes HCC development in several ways: it damages the integrity of the gut-vascular barrier (GVB), alters the tumor microenvironment (TME), and even affects the intratumoral microbiota. Subsequently, intratumoral microbiota present a characteristic profile and play an essential role in HCC progression mainly by causing DNA damage, mediating tumor-related signaling pathways, altering the TME, promoting HCC metastasis, or through other mechanisms. Both gut microbiota and intratumoral microbiota have dual effects on HCC progression; a comprehensive understanding of their complex biological roles will provide a theoretical foundation for potential clinical applications in HCC treatment.

## FACTS

1. Gut dysbiosis drives HCC progression through mechanisms such as gut-vascular barrier (GVB) disruption, tumor microenvironment (TME) remodeling, and translocation of microbial metabolites (e.g., LPS, SCFAs) that induce chronic inflammation and immune suppression.
2. TLR4 signaling is a central hub linking gut dysbiosis to HCC progression. LPS-TLR4 interactions drive stemness, angiogenesis, and metastasis through various signaling pathways, metabolites, and immune cells, highlighting its potential as a therapeutic target.
3. Bile acids and SCFAs mediate dual roles in HCC. While hydrophobic bile acids (e.g., DCA) promote HSC senescence and Treg expansion, SCFAs like butyrate suppress tumor growth via CD8^+^ T cell activation. However, acetate’s role is context-dependent, influenced by dietary factors and microbial sources.
4. Intratumoral microbiota exhibits HCC-specific profiles, with increased *Proteobacteria*, *Firmicutes*, and *Fusobacterium* in tumor tissues. These microbes are closely associated with gut microbiota and contribute to DNA damage, signaling pathway activation (e.g., TLR4/JNK), and immunosuppression.

## OPEN QUESTIONS

1. How do HCC etiologies (e.g., viral vs. non-viral) shape gut and intratumoral microbiota heterogeneity? Are microbial biomarkers (e.g., *Klebsiella* for microvascular invasion) universally applicable across subtypes?
2. What molecular pathways link microbial metabolites to epigenetic reprogramming (e.g., TET2 in B cells, FOXP3 in Tregs) and HCC progression? Can these pathways be reversed through dietary or pharmacological interventions to become therapeutic targets in the clinical treatment of HCC?
3. Since the relationship between the microbiota and the immune system is so intimate, can modulation of specific microbial taxa (e.g., *Akkermansia*, *Bifidobacterium*) or metabolites (e.g., butyrate) enhance immunotherapy efficacy (e.g., anti-PD-1) in HCC? What are the optimal strategies for microbiota-targeted adjuvant therapies?
4. What mechanisms govern the translocation and colonization of gut-derived microbes into HCC tissues? How do intratumoral microbiota interact with the TME to influence immune evasion or metastasis?

## Introduction

### Hepatocellular carcinoma

Hepatocellular carcinoma (HCC) is the most common type of liver malignancy, accounting for approximately 90% of cases, and one of the most common cancers worldwide. It is also one of the most harmful tumors, characterized by high morbidity and mortality rates [1, 2]. According to 2022 GLOBOCAN estimates, there were 865,269 new cases worldwide, accounting for 4.3% of the new cancer cases that year. Moreover, it is the third leading cause of cancer-related death globally, causing 757,948 deaths (7.8%) in 2022 [3].

The pathophysiology of HCC, from oncogenesis to progression, is a complex, multistep process involving dysregulation of cancer driver genes and related signaling pathways [4, 5], abnormal infiltration of immune cells [6, 7], metabolic factors (such as glucose, lipids, and key enzymes) [8–12], and many other factors. Among these factors, the gut microbiota plays a significant role.

### Gut microbiota

#### Gut microbiota composition

The human gastrointestinal tract harbors a complex ecosystem of approximately 2000 genera of bacteria, fungi, archaea, and viruses [13, 14]. The collective genomes of these microorganisms, known as the gut microbiome, encode an estimated five million genes [15, 16]. In this review, we concentrate primarily on the gut bacteria, highlighting the predominance of *Bacteroidetes* and *Firmicutes*, which together account for nearly 90% of the bacterial population. Other less dominant bacteria include *Proteobacteria* (*Pseudomonadota*), *Actinobacteria* (*Actinomycetota*), and *Verrucomicrobia* [17].

#### Gut microbiota function

The gut microbiota has important functions for the host. The microbes not only participate in food digestion, molecules decomposition and synthesis, and absorption, providing energy and nutritional support for the host [18, 19], but they also maintain the normal host-physiological functions by regulating metabolism [20–22]. By directly or indirectly interacting with the immune system and other aspects of the internal environment, the gut microbiota further protects the host against pathogen invasion and influences disease progression [23–25].

## Gut dysbiosis in HCC

Gut microbial dysbiosis is the imbalance or other similar alteration in the composition and/or function of the gut microbiota, which may lead to or worsen diseases [26, 27].

### The dysbiosis profile

Several studies have found that the gut microbiota in HCC is characterized by a distinctive dysbiosis profile [28]. The structure of the gut microbiota was found to be significantly altered in HCC patients compared to non-HCC patients with or without liver cirrhosis, while no significant difference in gut microbiome composition was found in HCC patients with different etiologies [29]. Across various studies involving different populations, fecal samples, and sample sizes, the trends in the gut dysbiosis profiles in HCC were concordant, though there were subtle differences in the exact results. For example, the phyla *Firmicutes* and *Actinobacteria* were significantly increased in the gut of HCC patients [30]. The genus *Clostridium* increased during HCC progression and may be related to bile acid metabolism [30, 31]. The genus *Ruminococcus* is greatly increased in patients with HCC compared to other liver diseases [32], yet this genus tends to colonize the intestines of HCC patients without liver microvascular invasion [33], which demonstrates the nuanced differences in gut microbiota distribution across HCC subtypes. The genera *Enterococcus* also tends to be increased in the gut of HCC patients [34, 35], including the *Enterobacter ludwigii*, which showed a significant increase of 100 times higher in HCC than other compared groups [36], and the *Enterococcaceae* which could be a potential marker for HCC diagnosis [37]. The genera *Streptococcus* and *Lactobacillus* belong to *Firmicutes* and enriched in the gut of HCC as well [38–40]. Although the genus *Bifidobacterium* mainly belongs to the phylum *Actinobacteria*, its abundance tends to be reduced in the gut of HCC patients as the beneficial bacteria [35], and decreases further as the disease progresses [34]. The genera belonging to the phylum *Actinobacteria* and increased in the gut of HCC patients include *Atopobium* [41] and *Gemmiger* [42]. In the phyla *Bacteroidetes*, fecal samples from HCC patients have higher levels of the genera *Desulfovibrio* [30], *Bacteroides* [30, 35], and *Parabacteroides* [42]. In the phyla *Proteobacteria*, levels of *Escherichia coli* [41] and *Shigella* [33] were increased as well.

Beneficial bacteria, typically reduced in the gut of HCC patients, include *Akkermansia*, *Prevotella_2*, *Subdoligranulum* and *Faecalibacterium* were decreased [38–40].

In a study held by Yang et al. [33] examining fecal microbiota profiles in 364 hepatitis B virus (HBV)-HCC patients and controls, *Streptococcus* (14.14% *v.s*. 5.67%) and *Escherichia-Shigella* (11.5% *v.s*. 9.55%) were more abundant in HCC than the control group, while *Agathobacter* was strongly decreased even though it belongs to the phylum *Firmicutes*. Li et al. [32] examined the gut microbiota in 68 patients with HCC, 33 patients with liver cirrhosis (LC), and 34 normal controls (NC). In their results, 21 genera, including *Roseburia, Lachnospira*, and *Ruminococcus*, were increased in HCC compared to LC, and 42 genera including *Veillonella*, *Faecalibacterium, Alistipes*, and *Phaecolarctobacterium*, were strongly decreased in HCC compared to NC. Nevertheless, 35 species, such as *Phocaeicola vulgatus*, *Lachnospira eligens*, *Bacteroides uniformis*, and *Ruminococcus bicirculans*, differed between HCC and LC. The other significantly increased species in HCC compared to NC included *Veillonella parvula*, *Veillonella* sp. T1–7, *Veillonella atypica*, and *Veillonella dispar*, and the decreased including *Phocaeicola dorei*, *Bacteroides uniformis*, *Faecalibacterium prausnitzii*, etc.

### Selective gut microbiota as potential biomarkers in HCC

Selective gut bacteria have emerged as potential biomarkers for HCC detection, demonstrating high sensitivity and specificity. For instance, *Odoribacter splanchnicus* and *Ruminococcus bicirculans* are potential species-level biomarkers of HCC [32], and the combinatorial detection of *Coriobacterium*, *Atopobium* and *Coprococcus* at the genus level was of high diagnostic value in HCC as well [43].

As biomarkers of HCC, the abundance of some gut bacteria closely changed with phenotypes during the tumorigenesis or progression. In a study by Ren et al. [42] analyzing fecal samples from patients, there are 13 genera including *Parabacteroides* and *Gemmiger* which were increased in early stage HCC and could serve as microbial markers for the early diagnosis of HCC. In a study by Zhang et al. [34] studying HCC patients as well, higher levels of *Enterococcus* and *Enterobacteriaceae* and lower levels of *Actinobacteria* and *Bifidobacterium* were statistically related to higher HCC stage and thus worse prognosis. The abovementioned *Streptococcus* and *Escherichia-Shigella* were more abundant in HCC and kept ascending during HCC progression thus had the potential of HCC biomarkers as well [33]. There were also significant differences in the gut microbiota between HCC patients with and without microvascular invasion (HCC-MVI or HCC-NVI), HCC-MVI is often accompanied by high invasiveness, high metastasis, and higher malignancy. In HCC-MVI compared to HCC-NVI, *Klebsiella*, *Proteobacteria*, *Prevotellaceae*, and *Enterobacteriaceae* were significantly enriched, whereas *Firmicutes*, *Ruminococcus*, and *Monoglobaceae* were significantly decreased. Among these gut bacteria, *Klebsiella* was the key biomarker that distinguished the two subgroups, even predicting clinical outcomes [44]. In 2019, Ni et al. [45] found that pro-inflammatory bacteria significantly increased in HCC patients compared to healthy controls. Furthermore, they introduced an assessment metric, the degree of dysbiosis (D(dys)), and found that it significantly increased in HCC patients compared to healthy controls. In addition, D(dys) increased consistently with HCC stage, although the differences among the stages were not significant.

**Fig. 1:**
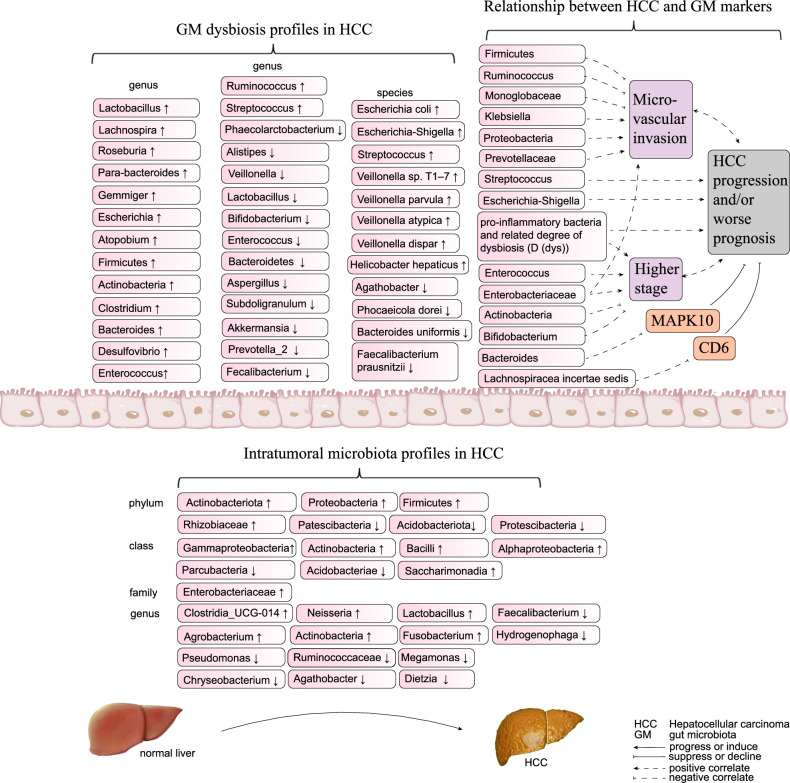
Gut microbiota (GM) and intratumoral microbiota profiles in hepatocellular carcinoma (HCC), and GM biomarkers associated with HCC progression.

In 2020, Huang et al. [31] collected fecal samples from 113 HBV-related HCC patients and 100 healthy controls for 16S rRNA sequencing, and selected 32 paired tumor and adjacent non-tumor liver tissues from the HCC group for next-generation RNA sequencing [31]. As a result, they found the increased abundance of *Lachnospiracea incertae sedis* was accompanied by a decrease in cluster of differentiation (cd) 6 expression, the increased abundance of *Bacteroides* was accompanied by a decrease in mitogen-activated protein kinase (mapk) 10 expression, and both of the bacteria were in line with sorrower disease-free survival (*P* = 0.0092 and 0.084, respectively). This result makes sense because CD6 and MAPK10 are tumor suppressors associated with better clinical prognosis. The profiles of gut microbiota and their biomarkers closely related with HCC progression are summarized in Fig. 1.

## Breakdown of gut-vascular barrier integrity due to gut dysbiosis

Foremost among all mechanisms, the initial biological event linking gut dysbiosis to tumor progression is the disruption of the gut-vascular barrier (GVB). The gut barrier consists of three layers. The outermost layer is the mucus layer, which mainly exists to shield against pathogens such as certain bacteria [46, 47]. To strongly decrease bacterial migration into the inner section of this mucus layer, antimicrobial peptides (e.g., defensins, lysozyme, and c-lectin Reg3γ) are secreted by intestinal epithelial cells and immune cells (Paneth cells), and immunoglobulin A (IgA) is secreted by plasma cells [48]. The middle layer consists of tightly connected epithelial cells, which ensure selective nutrient transfer while limiting the entry of gut microbes, especially pathogens, from the gut into the host [49]. The innermost layer is the blood vessel endothelium. If pathogens translocate through the intestinal epithelium into the bloodstream, they are mostly blocked by this layer and eliminated by circulating macrophages or engulfed by dendritic cells (DCs) before being transported to mesenteric lymph nodes [50].

In the healthy state, the GVB is characterized by the selective permeability of nutrients, metabolites, water, and bacterial products, the GVB integrity is governed by cellular, neural, immune, and hormonal factors [51]. While in the context of gut microbiota dysbiosis, the integrity of the GVB can be is compromised through a series of interconnected mechanisms. The disruption of the gut barrier, also known as increased intestinal permeability or “leaky gut,” is driven by several factors related with the gut microbiota dysbiosis, briefly including: (1) Inflammation: Chronic low-grade inflammation, often a result of dysbiosis, can lead to the release of pro-inflammatory cytokines and chemokines that damage the tight junctions between intestinal epithelial cells, thereby increasing permeability [51]. For example, HCC patients had a higher level of *Proteobacteria*, which favors intestinal mucosa inflammation and thus higher gut barrier permeability [52]. Another example, increased harmful microbiota approaching the epithelium can contribute to the local inflammation [53], and dysbiosis-caused increased populations of pathogenic species of bacteria like *E. coli*, *Bacteroides Enterococci* and *Clostridium histolyticum*, which could dismiss higher levels of lipopolysaccharides (LPS) damaging the enterocytes, thus disrupting GVB [54]. (2) Direct Invasion by Pathogens: Some bacteria associated with dysbiosis can invade the gut mucosa, causing damage to the epithelial layer or its function [51, 55]. (3) Metabolic Disruptions: Some products or metabolites which are secreted or regulated by the gut microbiota can affect the balance and function of the intestinal epithelium, leading to barrier dysfunction [56]. *Akkermansia*, which is decreased in HCC [35], protects the GVB by metabolites like acetate and propionate [57, 58]. This bacteria can also improve GVB by upregulating the expression of some TJ proteins or significantly reduce the LPS level [59].

As a result, gut dysbiosis increases the permeability of the barrier, which allows substances such as short-chain fatty acids (SCFAs), bile acids (BAs), bacterial constituents, choline, and endogenous ethanol to enter the liver, increasing the hepatic metabolic load and inflammatory stimulation, thus increasing the progression of HCC [60]. Due to increased intestinal permeability, factors such as viable pathogenic bacteria, Gram-negative microbial products, and pro-inflammatory luminal metabolites can translocate from the intestinal lumen to the liver via the portal circulation and thereby alter the hepatic microenvironment [49, 61].

## Mechanisms by which the gut microbiota influences the HCC-related microenvironment

The gut microbiota can profoundly affect the immune microenvironment of the liver and thereby trigger hepatocarcinogenesis via the following processes: (1) microbial activation of signaling like Toll-like receptor (TLR)-4; (2) microbial stimulation of tumor microenvironment (TME) cells; and (3) influence of gut microbiota-associated metabolites. The typical examples are summarized in Table 1.

**Table 1:** Modulation of gut microbiota and related downstream molecules in HCC promotion.

Gut microbiota	Year	Effect	Mechanism	Level of evidence
TLR4 in liver cancer cells	2012	Promoted intrahepatic metastasis	Induced the recruitment of Tregs and interacted with TAMs	In cell lines [75].
*Lactobacillus reuteri* FLRE5K1, isolated from the liver	2012	Inhibited HCC tumor formation and growth	Activated the IFN-γ/CXCL10/CXCR3 pathway, stimulated Th1 cells differentiation and inhibited Tregs differentiation	In a diethylnitrosamine (DEN) model of rat hepatocarcinogenesis [41].
A novel probiotic mixture Prohep (comprising *Lactobacillus rhamnosus GG*, *E. coli Nissle 1917* and *heat-inactivated VSL#3*)	2016	Slowed down the tumor growth significantly and reduced the tumor size and weight by 40% compared with the control.	Decreased Th17 cells, increased certain beneficial bacteria like *Prevotella* and *Oscillibacter* for anti-inflammation effect, thus mediated the T-cell differentiation against HCC.	In mouse model [211].
Hydrophobic bile acids like DCA-, LCA-, or TCDCA and bile acid-modulating bacteria	2016	Promoted hepatocarcinogenesis	Made HepG2 cells grow faster, exerted higher level of c-Myc, and lower level of CEBPα.	In NASH-HCC mouse model and HepG2 lines [30].
Lipoteichoic acid (LTA), a cell wall component of gut microbiota	2017	Impaired the anti-tumor function of T cells and contribute to HCC progression	LTA-TLR2-COX2- PGE_2_ axis upregulated numbers of Tregs and PD-1^+^ CD8^+^ T cells by binding to receptor PTGER4.	In obesity-induced HCC mouse model [118].
Clearance of bile acid-modulating bacteria like *C. scindens*	2018	NKT cells were recruited and procedured anti-tumor function	Bile acid- CXCL16-CXCR6 axis.	In mouse model reported in 2018 [101],
TLR4 in liver cancer cells	2019	Induced the cancer stem cells (CSCs) of HCC	LPS activated TLR4-AKT-SOX2 signaling pathway	In cell lines [72].
IL-25 secreted by hyperplastic epithelial tuft cells in colon and induced by gut microbiota dysbiosis.	2019	Promoted HCC cell migration, invasion and tumorigenesis.	IL-25 increased the M2 percentage (CD206/CD68) and CXCL10 secretion of macrophages to promote HCC.	Mainly cell lines and vitro study [96].
TLR4 in liver cancer cells	2020	Improved HCC angiogenesis and pulmonary metastasis	TLR4-MyD88 axis activated STAT3/Sp1-dependent VEGF overexpression	Orthotopic HCC-implanted mice model [65].
TLR4 in hepatic progenitor cells (HPCs)	2020	Increased the susceptibility of HPCs to malignant proliferation	Promoted fibrotic differentiation of HPCs and enhanced the production of IL-6 and TNF-α thus influenced Ras and p53 signaling pathways.	In rat model transplanted with exogenous HPCs and induced to HCC by diethylnitrosamine, [73].
SCFAs treatment (consisting of the sodium salts of butyrate, propionate and acetate),	2021	Delayed the progression of HBV-HCC,	Rescued the altered signaling pathways affected by HBx, including Ras and NF-κB signaling. Increased expression of DAB2 thus depressed Ras pathway activity	In HBx transgenic (HBxTg) mice model [146].
TLR4 in tumor-associated neutrophils	2022	Promoted alcoholic HCC development.	Gut microbiota induced LPS-TLR4 promoted Protumorigenic N2 TANs and related NETs.	In mouse model of alcoholic HCC [76].
*Akkermansia muciniphila*	2022	Prevent NASH-associated HCC.	Increased hepatic CXCR6^+^ natural killer T (NKT) cells and decreased macrophage infiltration.	In mouse model of NASH-associated HCC and HepG2 cell lines [102].
*Lactobacillus*	2022	Improved HCC progression and prognosis.	Controlled specific gut microbiota composition, modulated MMP9 and NOTCH 1 signaling pathways through the interaction of gut microbiota and BA.	In a mouse model with T2DM and HCC [212].
Lipoteichoic acid (LTA), a cell wall component of gut microbiota	2022	Promote HCC development via the activation of Treg cells	Trigger IL-33 and IL-1β to release from senescent HSCs	HCC mouse model induced by high-fat diet [117].
*Lactobacillus reuteri*	2023	Slow down HCC progression and enhanced the efficacy of HCC immunotherapy.	Increased acetate (SCFA) levels, to reduce the production of IL-17A in hepatic type 3 innate lymphoid cells, thus exerting anti-tumor effect.	In mice model of HCC [147].
*Bifidobacterium pseudolongum*-generated acetate	2023	Restrained NAFLD-HCC formation and progression.	Bounded to G coupled-protein receptor 43 (GPR43) on hepatocytes in liver. GPR43 activation suppressed the IL-6/JAK1/STAT3 signaling pathway.	In two mouse models of NAFLD-HCC: diethylnitrosamine + a high-fat/high-cholesterol diet or + a choline-deficient/high-fat diet. Germ-free mice were used for the metabolic study of gut microbiota [148].
Gut microbiota-derived acetate induced by high dietary fructose	2023	Upregulated uridine diphospho-N-acetylglucosamine (UDP-GlcNAc) and enhanced protein O-GlcNAcylation in HCC, thus promoted HCC progression.	Acetate was converted from fructose by the gut microbiota and then served as the major acetyl-CoA donor in liver [150], which increased glutamine synthesis thus higher O-GlcNAcylation in fructose-rich environment, hyper-O-GlcNAcylation of eukaryotic elongation factor 1A1 (eEF1A1) promotes cell proliferation and tumor growth [149].	In wild-type C57BL/6 mice using a spontaneous and chemically induced HCC mouse model [149].

*TLR* toll-like receptor, *TAMs* tumor-associated macrophages, *HCC* hepatocellular carcinoma, *NASH* non-alcoholic steatohepatitis, *Tregs* regulatory T cells, *DCA* deoxycholic acid, *LCA* lithocholic acid, *TCDCA* taurochenodeoxycholate, *SCFA* short-chain fatty acid, *TANs* tumor-associated neutrophils, *NETs* neutrophil extracellular traps, *BA* bile acid, *T2DM* type 2 diabetes mellitus.

### PAMP-PRR (TLR4) related signal pathways

Pattern-recognition receptors (PRRs) can sense gut microorganisms based on their pathogen-associated molecular patterns (PAMPs; namely, “microbe-associated molecular patterns”, MAMPs) and subsequently exert biological effects. Among the PRRs, TLR4 is an important PRR that plays an immunosuppressive role and it is a key contributor to hepatocarcinogenesis [62, 63].

TLR4 is widely expressed in liver cancer stem cells, hepatocytes, Kupffer cells, hematopoietic stem cells, DCs, natural killer (NK) cells, B cells, and T cells. TLR4 predominantly recognizes LPS from Gram-negative bacteria [63–66] and is downregulated by HCC-suppressing microRNAs (miRNAs) such as miR122 [67]. Gut dysbiosis and TLR4 (and associated signaling) are required not for the initiation of HCC, but for its development [68].

TLR4 is overexpressed in HCC [69] and correlates with microvascular invasion, early recurrence, and poor prognosis in HCC patients [66, 70, 71].

In early HCC patients, LPS-producing gut bacterial genera and LPS were both increased [42]. LPS activated the TLR4-AKT-SOX2 signaling pathway and thereby induced HCC stem cells [72]. Furthermore, LPS/TLR4 signaling promoted HPC fibrotic differentiation and increased interleukin (IL)-6 and tumor necrosis factor (TNF)-α production in rats transplanted with diethylnitrosamine-induced HCC and with exogenous hepatic progenitor cells (HPCs) [73].

In another study, TLR4 recognized LPS and directly activated c-Jun N-terminal kinase (JNK)/MAPK signaling to improve epithelial-mesenchymal transition (EMT), tumor cell invasion, and metastasis [74]. Furthermore, the TLR4-MyD88 (namely Myeloid Differentiation Primary Response 88) axis activated STAT3 (Signal Transducer and Activator of Transcription 3) and SP1 (Specificity Protein 1), which are both transcription factors of the mRNA of the vascular endothelial growth factor (VEGF), so VEGF was overexpressed, which improved HCC angiogenesis and pulmonary metastasis [65].

In addition to directly affecting tumor cells, TLRs modulate other immune cells or stromal cells to affect the HCC TME. Dysbiotic microbiota in Nlrp6^(−/−)^ mice (the mice with mutant NLR family, pyrin domain containing 6) induces a TLR4-dependent expansion of hepatic monocytic myeloid-derived suppressor cells (mMDSCs) and suppresses T-cell abundance. This phenotype is transmissible via fecal microbiota transfer and reversed by antibiotics, pointing to the high plasticity of the TME. While the loss of *Akkermansia muciniphila* correlates with mMDSC abundance, its reintroduction restores intestinal barrier function and strongly reduces liver inflammation and fibrosis. Furthermore, a study showed that cirrhosis patients have increased bacterial abundance in hepatic tissue, which induces pronounced transcriptional changes, including activation of fibro-inflammatory pathways and cancer immunosuppression processes. This study demonstrated that the gut microbiota closely shapes the hepatic inflammatory microenvironment, offering new approaches for cancer prevention and therapy. TLR4 seemed to indirectly manage the recruitment of regulatory T cells (Tregs) and interacted with macrophages to promote intrahepatic metastasis [75]. TLR4 induced neutrophil extracellular traps (NETs) in the liver and thereby promoted alcoholic steatosis and, eventually, alcoholic HCC [76], it increased HCC metastasis potential mainly bypassing tumorous inflammatory response [77].

Other signaling pathways downstream of the gut microbiota exerted more complicated and even dual effects. For example, certain nonpathogenic *E. coli* strains stimulated the transcription of genes related to nuclear factor kappa-light-chain-enhancer of activated B cells (NF-κB) mediated inflammatory signaling pathways, while upregulated negative feedback regulators in nucleotide-binding oligomerization domain (NOD)-like signaling pathways, such as tumor necrosis factor alpha-induced protein 3 (TNFAIP3), tempered the inflammatory reaction [78]. Understanding whether and how these factors influence HCC development requires further exploration.

### HCC-associated cells and TME

**Fig. 2:**
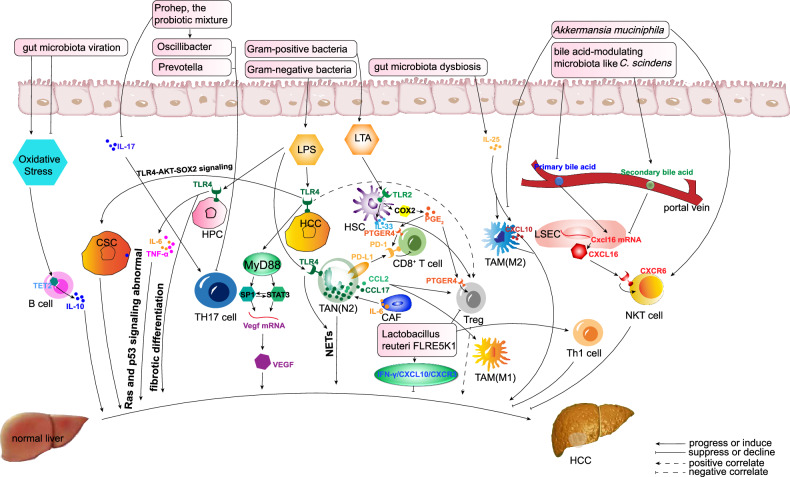
The gut microbiota influences HCC progression through the interaction with molecular signaling pathways and various cells. LPS/TLR4 signaling (activated by Gram-negative bacteria) promotes fibrotic differentiation of hepatic progenitor cells (HPCs) and enhances IL-6/TNF-α production, disrupting Ras and p53 pathways to drive malignant proliferation. The TLR4-MyD88 axis activates STAT3 and SP1, upregulating VEGF expression and promoting angiogenesis/metastasis. *C. scindens* converts primary bile acids into secondary bile acids, decreasing CXCL16 expression and NKT cell recruitment. LTA from Gram-positive bacteria activates IL-33/IL-1β in hepatic stellate cells (HSCs) via TLR2, promoting Treg-mediated HCC progression.

The TME is a complex integrated system mainly composed of tumor cells, surrounding immune and inflammatory cells, tumor-related fibroblasts, nearby interstitial tissues, microvessels, and various cytokines and chemokines [79, 80]. Gut microbes and their metabolites can influence HCC pathogenesis and progression by changing the intestinal microenvironment, altering ligands for specific receptors and modulators of certain protein activities, modulating signaling pathways, and ultimately influencing the expression of various genes in key cells and multiple cytokine levels in the TME [64, 81]. How the gut microbiota influenced HCC progression through the interaction with molecular signaling pathways and various cells were summarized in Fig. 2.

In addition to hepatocytes and HCC cells, the HCC TME involves the following cells: (1) immune cells, such as neutrophils, circulating monocytes/macrophages, resident macrophages (Kupffer cells), DCs, NK cells, natural killer T (NKT) cells, B cells, and T cells such as CD8^+^ T cells, CD4^+^ T cells, Tregs, and γδ T cells [82] and (2) other liver-resident cells in the environment or stroma surrounding the HCC cells, such as hepatic stellate cells (HSCs), fibroblasts, and liver sinusoidal endothelial cells [83, 84].

#### Neutrophils

Tumor-associated neutrophils (TANs) in HCC can be categorized as antitumorigenic (N1) or pro-tumorigenic (N2) [85]. Pro-tumorigenic N2 TANs support HCC by forming decondensed chromatin embedded with granular proteins, namely NETs [85]. Gut microbiota-induced LPS-TLR4 signaling promoted pro-tumorigenic N2 TANs and related NETs in alcoholic HCC [76]. During HCC treatment, TANs were reported to recruit tumor-associated macrophages and Tregs through the secretion of chemokine (C-C motif) Ligand (CCL) 2 and CCL17 to induce drug resistance [86].

#### Macrophages

Macrophages are classified into two functionally and phenotypically distinct categories: macrophages with an embryonic origin that reside in the liver after specific differentiation (Kupffer cells), and macrophages differentiated from circulating monocytes (circulating monocyte-derived macrophages, called “macrophages” below for short) [87]. When circulating macrophages are recruited to the liver via stimulation by pathological factors in the TME, they polarize into different phenotypes (mainly M1 and M2) [88]. M1 macrophages have an antigen-presenting function, so they can combat pathogenic microorganisms [89] and tumor formation [90], exerting pro-inflammatory effects [91]. M2 macrophages usually support anti-inflammation effects [91], tissue remodeling and angiogenesis [92, 93], while promoting tumors [94]. The gut microbiota can alter macrophage polarization by stimulating certain signaling pathways in the gut microenvironment. In a mouse model of colon inflammation, deoxycholic acid (DCA) was enriched by increased levels of Gram-positive bacteria, which promoted macrophage polarization toward the pro-inflammatory M1 phenotype (partially through TLR2 transactivated by the M2 muscarinic acetylcholine receptor) [95]. However, there was no concrete evidence that bile acids regulated HCC via these mechanisms.

When gut dysbiosis promoted M2 macrophage polarization, HCC pathogenesis was promoted. For example, IL-25, which was secreted by colonic hyperplastic epithelial tuft cells and induced by gut dysbiosis, increased the M2 percentage (CD206/CD68) and Chemokine (C-X-C motif) Ligand (CXCL) 10 secretion of macrophages to promote HCC [96]. Macrophages also contributed to HCC subcomponent phenotypes. A study in 2022 found an increased proportion of M2-type tumor-associated macrophages in HCC patients with microvascular invasion compared to HCC patients without microvascular invasion (*P* < 0.001). The exact mechanism underlying this phenomenon needs to be further explored [44].

#### Kupffer cells

Kupffer cells are the key cells that interact with LPS in the liver, and they also play a key role in inducing cytokines such as TNFα and IL-6, which promote the development of HCC associated with microbe-related LPS-TLR signaling [97]. In addition to HCC, Kupffer cells can affect other liver diseases. For example, Kupffer cells ameliorate early alcohol-induced liver injury after endotoxin activation via TLR4 [98]. In an animal model of non-alcoholic steatohepatitis (NASH), various factors such as metabolic disorders, oxidative stress, and translocated bacterial products activated Kupffer cells via TLRs, especially TLR4, leading to increased NF-κB signaling and pro-inflammatory cytokine production [99]. When translocated bacterial DNA bound to TLR9 on Kupffer cells, the cells produced IL-1b, thereby stimulating hepatocytes to accumulate lipids and inducing liver fibrosis [100].

#### NKT cells

In a mouse model reported in 2018 [101], NKT cell accumulation was regulated by CXCL16 from liver sinusoidal endothelial cells, which was controlled by gut microbiome-mediated primary-to-secondary bile acid conversion. For example, bile acid-modulating commensal bacteria like *C. scindens*, could convert primary bile acids into secondary bile acids. While antibiotics significantly increased primary bile acids, tauro-β-muricholic acid (T-β-MCA) and β-MCA, and decreased secondary bile acids, T-ω-MCA, taurodeoxycholic acid, ω-MCA, taurolithocholic acid, and tauroursodeoxycholic acid. Secondary bile acid ω-MCA decreased cxcl16 mRNA expression, whereas the primary bile acid T-β-MCA induced Cxcl16 mRNA. As a result, antibiotics indirectly upregulated CXCL16 in liver sinusoidal endothelial cells. Next, the accumulated CXCL16 bound to C-X-C Chemokine Receptor (CXCR) 6 on NKT cells and thereby recruited them, which led to anti-tumor effects against HCC. In contrast, bile acid-modulating bacteria (such as *C. scindens*) induced HCC progression via the bile acid-CXCL16-CXCR6 axis. In a NASH-associated HCC mouse model, NKT cells were stimulated by the gut microbe *A. muciniphila*, which thereby prevented HCC progression [102].

#### B cells

B cells contribute to NASH pathogenesis by activating metabolic T cells and monocyte-derived macrophages (via IgA secretion), thereby promoting fibrosis [103].

In the HCC TME, B cells usually interact with T cells to enhance anti-tumor effects thus improving prognosis [104]. For instance, B cells could be triggered by oxidative stress and then activate Tet methylcytosine dioxygenase 2 (TET2) to promote IL-10 expression [105], and high levels of IL-10 in B cells are usually correlated with HCC progression [106]. Theoretically, gut microbiota could regulate the TET2 level in B cells by modulating oxidative stress [107], and TET2 downregulation supported antitumor immunity to improve anti-programmed death (PD)-1 treatment for HCC [105]. In this way, TET2 inhibition in B cells by the gut microbiota may aid HCC treatment.

#### T cells

In the pathogenesis of HCC, the gut microbiota and their metabolites often interact with Tregs to establish immunosuppression in the liver [108].

In 2021, nonalcoholic fatty liver disease (NAFLD)-HCC was characterized by expansion of *Bacteroides caecimuris* (*P* < 0.0001) and *Veillonella parvula* (*P* = 0.002) and dysbiosis of other gut microbes [28]. Accordingly, flow cytometry showed that NAFLD-HCC induced the recruitment of effector IL-10^+^ Tregs, thus attenuating the expansion of cytotoxic CD8^+^ T cells [28]. Both Tregs and CD8 + T cells in HCC TME expressed dysfunction markers such as PD-1, Lag-3, and Tim-3 [109].

Many studies have determined the critical role of CD8^+^T cells in defending against HCC initiation and progression, which improves prognosis [110]. Different CD8 + T cells may predict different HCC incidences. CD8^+^ resident memory T cells and Tregs were enriched in HBV-related HCC, whereas Tim-3^+^CD8^+^ T cells and CD244^+^ NK cells were enriched in non-viral-related HCC [111]. CD8^+^T cells were recently found to predict truly recurrent HCC [112]. Single-cell RNA-sequencing of 34 samples from 20 recurrent HCC patients showed that the TME of truly recurrent HCC had more KLRB1 (Killer Cell Lectin Like Receptor B1, namely CD161)^+^CD8^+^ T cells with memory phenotype and low cytotoxicity, while the TME of de novo recurrent HCC had more cytotoxic and exhausted CD8^+^ T cells [112].

CD8^+^ T cells are generally considered to have anti-tumor effects, while expression of other different markers would present different states and functions of T cells, thus may exert totally different effects on HCC. For example, in the pro-inflammation and pro-tumoral environment formed by the gut microbiota, CD8^+^CXCR6^+^PD1^+^ T cells usually have an auto-aggressive, exhaustive and resident phenotype, which ultimately contribute to HCC pathogenesis [113].

With the effect of inhibiting the proliferation, activation and functions of other cytotoxic T cells, Foxp3^+^ CD4^+^ regulatory T cells (Tregs) became the most crucial subset of CD4^+^ T cells during HCC progression, especially from simple steatosis to HCC for their immunosuppressive effects [108]. Deoxycholic acid (DCA) and lithocholic acid (LCA) were the most abundant metabolites of the gut microbiota, and some gut microbiota may synthesize certain molecules of the bile acids [114]. Certain LCA derivative, isoalloLCA, was found to stimulate mitochondrial reactive oxygen species to upregulate FOXP3, thus exerting an immunosuppressive effect by inducing Treg differentiation [115]. 3β-hydroxydeoxycholic acid (isoDCA) also induced Treg differentiation by acting on DCs to activate FOXP3 [116]. Some subtypes of LCA or DCA synthesized by the gut microbiota may also mediate T cells through certain mechanisms.

#### HSCs

In a mouse model of HCC induced by a high-fat diet [117], lipoteichoic acid (a cell wall component of Gram-positive gut microbes) triggered IL-33 and IL-1β released from senescent HSCs. IL-33 was IL-1β-dependent and promoted HCC development via Treg activation in the TME. In 2017, a mouse model was used to show that obesity-induced lipoteichoic acid suppressed antitumor immunity and thereby promoted HCC [118]. The lipoteichoic acid enhanced the senescence-associated secretory phenotype and the COX2 level in HSCs in a process involving TLR2. As a result, COX2-mediated prostaglandin E2 (PGE2) boosted Tregs and PD-1^+^ CD8^+^ T cells by binding to PTGER4 (Prostaglandin E Receptor 4) on them, thereby contributing to HCC progression. Tregs can upregulate PD-1 on CD8^+^ T cells, and PD-1 is an inhibitory molecule that impairs the antitumor effect of T cells [119]. COX2 overexpression and excess PGE2 production were detected in HSCs in human NASH-triggered HCC, suggesting that the gut microbiota-driven COX2-PGE2-PTGER4 pathway may function in human NASH-associated HCC [118].

#### Fibroblasts

In HCC progression, cancer-associated fibroblasts induced neutrophil chemotaxis, protected neutrophils from spontaneous apoptosis, and activated neutrophils via the IL6-STAT3 signaling cascade, and then these neutrophils impaired T cell function via the Prostaglandin E Receptor 41/PD-L1 signaling pathway [120].

### Microbe-derived metabolites mediate HCC progression

#### Bile acids

Bile acids are key metabolites related to gut bacteria or the whole gut microbiota [115]. They are synthesized from amphipathic cholesterol in the liver and directly regulated by the farnesoid X receptor (FXR)-fibroblast growth factor 19 (FGF19) axis [121] and G protein-coupled bile acid receptor 1 (GPBAR1/TGR5) [122]. Bile acids are modulated by gut bacteria to generate bioactive molecules [115], with the major effect of solubilizing dietary lipids to help their absorption in the small intestine [123]. After that, about 95% of bile acids are reabsorbed and recycled via the gut–liver axis, and the remaining 5% of bile acids enter the colon to facilitate the production of the major serum bile acids, DCA and LCA, via dihydroxylation; this process is mediated by gut microbes [3, 124, 125].

One of the most important biological effects of bile acids is their crucial role in the differentiation of T cells and macrophage polarization. Distinct derivatives of LCA and DCA (including iso-, 3-oxo-LCA/DCA, allo-, 3-oxoallo-, and isoalloLCA) are involved [126, 127]. 3-oxoLCA inhibited the differentiation of Th17 cells by binding to the small molecule agonist RORγt [115], while isoalloLCA stimulated anti-inflammatory Treg differentiation by upregulating FOXP3 via mitochondrial reactive oxygen species generation and the binding of NR4A1 at the Foxp3 locus [128]. 3β-hydroxydeoxycholic acid (isoDCA) also induced Treg differentiation by acting on DCs to activate FOXP3 [116]. In a mouse model of colonic inflammation, DCA was enriched after Gram-positive bacteria increased, and DCA then promoted macrophage polarization toward the pro-inflammatory M1 phenotype partially via TLR2 transactivated by the M2 muscarinic acetylcholine receptor [95]. As T cells and macrophages both affect the hepatocarcinogenesis-related immune response, bile acids may regulate HCC via these mechanisms.

In recent years, there has been increasing evidence that the gut microbiota influences hepatic metabolic homeostasis by regulating bile acid synthesis and metabolism, leading to HCC development and progression.

DCA, a hydrophobic bile acid, has been reported to be closely related to DNA damage and cell survival [129, 130]. In mice, dietary or genetic obesity increased the DCA level (related to dysbiosis). DCA then induced HSC senescence, thus stimulating the secretion of multiple cytokines that induce hepatocarcinogenesis [131].

Hydrophobic bile acids, under the control of the gut microbiota, may promote HCC. During NASH-HCC formation and development, when bacterial taxa involved in bile acid metabolism (such as the genera Clostridium, Bacteroides, and Desulfovibrio) were significantly increased, the taurochenodeoxycholate (TCDCA) level was strongly increased, while decreasing hydrophobic bile acids reversed NASH-HCC progression [30]. In further in vitro tests, DCA-, LCA-, and TCDCA-treated HepG2 cells all grew faster and had higher oncoprotein levels, illustrating the hepatocarcinogenesis-promoting effects of these hydrophobic bile acids [30]. These findings were further supported by Ma et al. in 2018 [101]. They found that gut microbiota-mediated primary-to-secondary bile acid conversion indirectly disturbed NKT cell accumulation and thereby interfered with HCC-related immune responses via the CXCL16-CXCR6 axis.

In 2020, Huang et al. [31] found that several groups of bile acid-associated gut microbes (*Bacteroides*, *Lachnospiracea incertae sedis*, and *Clostridium XIVa*) were related to transcriptome changes in the TME in HBV-related HCC patients, suggesting that bile acids may be important mediators of the communication between the gut microbiota and HCC, but the mechanisms need further study. Furthermore, in 2023, Li et al. [32] found that taurochenodeoxycholic acid and glycochenodeoxycholate were both tightly associated with HCC in their cohort study, indicating the potential diagnostic value of secondary bile acids in HCC.

The liver not only regulates bile acid production and enterohepatic circulation, but also absorbs nutrients from the portal vein and metabolites from the gut microbiota [132], thus regulating lipid and glucose metabolism [133]. The gut microbiota complements the host with additional metabolic functions including the digestion of complex polysaccharides [134, 135] and the production of fatty acids [136, 137], amino acids [138], and vitamins [139]. The gut bacteria have the genetic potential to perform thousands more chemical reactions than humans [140]. SCFAs are metabolites that are controlled by the gut microbiota and have the greatest impact on HCC.

#### SCFAs

Several studies have found that the gut microbiota in HCC is characterized by a distinctive dysbiosis profile [28]. During HCC development, the gut microbiota supports SCFA production and elicits a T cell immunosuppressive phenotype. SCFAs, which are produced by the anaerobic fermentation of dietary fiber by the gut microbiota, are characteristically altered during the pathogenesis of HCC, and greatly influence tumor immunity.

For example, in a study in 2021, the gut microbiota of NAFLD-HCC patients compared to patients with other liver diseases was characterized by expansion of Proteobacteria at the phylum level, and expansion of *Enterobacteriaceae* and reduction of *Oscillospiraceae* and *Erysipelotrichaceae* at the family level. At the species level, *Bacteroides caecimuris* (*P* < 0.0001) and *Veillonella parvula* (*P* = 0.002) were significantly enriched in the NAFLD-HCC patients compared to the NAFLD-cirrhosis patients and non-NAFLD controls [28].

Furthermore, genes related to acetate and butyrate/acetylphosphate synthesis were both overexpressed in the NAFLD-HCC patients compared to the others [28]. Accordingly, Oxaloacetate and acetylphosphate, crucial SCFA intermediates, were significantly elevated in the feces of the NAFLD-HCC patients [28]. The feces of the NAFLD-HCC patients had characteristically higher levels of SCFAs such as acetate, butyrate, and formate (*P* < 0.0001 for all) compared to the NAFLD-cirrhosis patients and non-NAFLD controls. In the serum, the increased SCFAs were butyrate (*P* = 0.005) and propionate (*P* = 0.0002) [28]. Based on statistical analysis of patient data, the gut microbes related to NAFLD-HCC seemed to activate the expansion of effector IL-10^+^ Tregs and restrain the expansion of cytotoxic CD8^+^ T cells partially via the effects of SCFAs [28].

HCC development and the associated immune response involves the participation of many T cells, and many studies have identified a potential role of SCFAs in mediating the biological effects of the gut microbiota on T-cell immunity [141–143]. Dysbiosis leads to the generation of excessive amounts of SCFAs. SCFAs, especially propionate, reduced IL-17 and IL-22 production by intestinal γδ T cells, and thus had the potential to suppress anti-tumor immunology [142]. On the contrary, Butyrate can be used in the tricarboxylic acid cycle (rather than relying on glycolytic input) in CD8^+^ T cells, so the cells preferentially exhibit oxidative phosphorylation. This increases CD8^+^ T cell activity and their long-term survival as memory cells, thus theoretically protect anti-tumor immunology [143]. Some SCFAs (under the control of the gut microbiota) have been reported to promote an immunosuppressive response in an anti-inflammatory environment by strongly regulating Tregs and CD8 + T cells in other diseases [143–145], suggesting the complex role of these SCFAs in HCC.

Several studies have reported the anti-HCC role of SCFAs. In an HBx (HBV-encoded oncoprotein) transgenic (HBxTg) mouse model fed SCFAs (consisting of the sodium salts of butyrate, propionate, and acetate), HCC foci were reduced and the altered signaling pathways affected by HBx were rescued, including those involving inflammation, phosphatidylinositol 3-kinase, epidermal growth factor, Ras, and NF-κB signaling. SCFAs also dose-dependently reduced HBx-transfected cell viability, suggesting that SCFAs may delay the progression of HBV-HCC, but the detailed mechanisms remain unclear. One of the mechanisms may involve SCFAs downregulating disabled homolog 2 (DAB2) and thus depressing Ras pathway activity [146].

In a mouse model of HCC, *Lactobacillus reuteri* was markedly reduced, together with decreased SCFAs levels, especially acetate. Further research found that *L. reuteri* could increase acetate levels, and acetate then reduced the production of IL-17A in hepatic type 3 innate lymphoid cells (ILC3), thus exerting an anti-tumor effect. Moreover, the combination of acetate with PD-1/PD-L1 blockade significantly enhanced antitumor immunity [147]. This implied that SCFAs such as acetate may not only be regulated by the gut microbiota and affect HCC by influencing T cell-associated immune effects, but may also influence HCC treatment by influencing immune checkpoint inhibitors.

Acetate seems to be a double-edged sword in the HCC process, different sources of acetate may have totally opposite effects. Song et al. recently found that acetate generated by *Bifidobacterium pseudolongum* suppressed NAFLD-HCC in two mouse models and NAFLD-HCC cell lines [148]. *B*. *pseudolongum* was the most depleted bacterium in mice with NAFLD-HCC, and *B*. *pseudolongum* supplementation significantly suppressed NAFLD-HCC formation. Acetate was verified as the crucial metabolite generated by *B*. *pseudolongum*. It bound to G protein-coupled receptor 43 (GPR43) on hepatocytes in the liver. GPR43 activation suppressed the IL-6/JAK1/STAT3 signaling pathway to restrain NAFLD-HCC formation and progression. However, in the same year in mouse models, Zhou et al. found that gut microbiota-derived acetate induced by high dietary fructose upregulated uridine diphospho-N-acetylglucosamine (UDP-GlcNAc) and enhanced protein O-GlcNAcylation in HCC, thus promoting HCC progression [149]. The mechanism involved acetate produced from fructose by the gut microbiota. The acetate served as a major hepatic acetyl-CoA donor [150], which increased glutamine synthesis, leading to higher O-GlcNAcylation in fructose-rich environments. Hyper-O-GlcNAcylation of eukaryotic elongation factor 1A1 (eEF1A1) then promoted cell proliferation and tumor growth [149].

Butyrate has antitumor properties. In HCC patients, the abundance of butyric acid-producing gut bacterial genera was decreased, butyrate metabolism was activated, and plasma butyrate levels were decreased [42]. Butyrate supplementation can not only inhibit HCC proliferation and metastasis, but also improve the anticancer efficacy of sorafenib by regulating intracellular calcium homeostasis [151] or affecting sorafenib-targeted miRNAs [152]. Butyric acid also plays a crucial role in the synergistic anti-HCC effect of peroxisome proliferator-activated receptor (PPAR)-δ and berberine. Berberine suppressed HCC, which was dependent on the binding of PPARδ to the promoters of apoptotic genes such as caspase 3, B-Cell CLL/Lymphoma 2 (BCL2) associated X protein (BAX), and BCL2. Butyric acid enhanced the efficacy of berberine by reducing PPARδ degradation via inhibiting the ubiquitin–proteasome system [153].

**Fig. 3:**
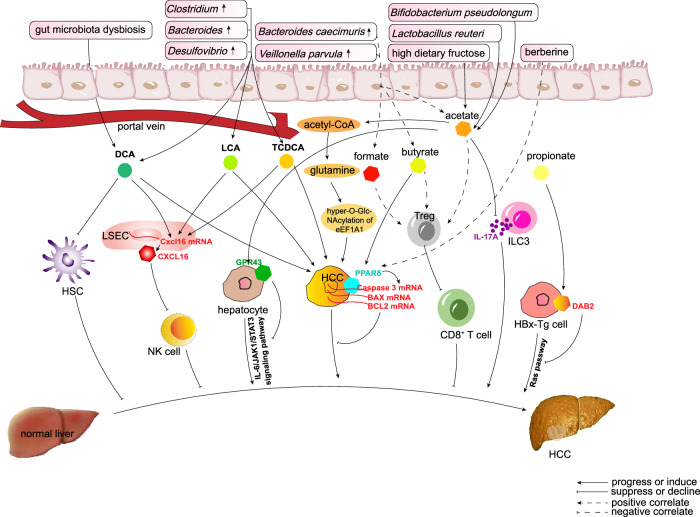
The gut microbiota modulates HCC progression via metabolites. SCFAs (butyrate, propionate, acetate) downregulate PI3K/NF-κB signaling in HBV-HCC. *L. reuteri*-derived acetate suppresses IL-17A in ILC3s, exerting anti-tumor effects. *Bifidobacterium pseudolongum*-derived acetate binds GPR43, inhibiting IL-6/JAK1/STAT3 in NAFLD-HCC. Butyrate synergizes with berberine to stabilize PPARδ, enhancing apoptosis via caspase 3/BAX.

As summarized in Fig. 3, gut microbiota derived metabolites usually have two-sided effects on modulating HCC progression.

## From microenvironmental imbalance to HCC

As indicated above, gut dysbiosis promotes HCC development through multiple mechanisms: altering PRR expression (e.g., TLRs) and downstream signaling, disrupting immune cell function, and inducing gut-liver metabolic dysregulation. These processes drive chronic inflammation, immune microenvironment remodeling, and immune evasion. For example, during NAFLD-cirrhosis-to-HCC progression, dysbiosis-induced SCFA accumulation expands IL-10^+^ Tregs and suppresses cytotoxic CD8^+^ T cells, fostering immunosuppression and hepatocarcinogenesis [28].

Enterobacterial dysbiosis alters the intestinal environment and further exacerbates enterobacterial dysbiosis by changing the immune microenvironment, thus creating a vicious cycle. *Enterobacteriaceae*, including *Klebsiella*, *E. coli*, *Aspergillus*, and *Enterobacteriaceae*, are among many potential pathogens. An overabundance of *Enterobacteriaceae* in the gut releases large amounts of LPS, which is positively correlated with upregulated serum IL-6, IL-1, and TNF-a [154]. These pathological changes may exacerbate enterobacterial dysbiosis by increasing transcellular permeability, thus impairing intestinal barrier function [155]. This ultimately exacerbates *Enterobacteriaceae*, *Haemophilus*, and *Enterococc*i overgrowth.

Regarding T cells and intestinal bacteria imbalance, gut dysbiosis and metabolic disorders usually lead to an intensely inflammatory environment in the liver [156], involving immune cells such as CD8^+^ T cells and NKT cells in HCC pathogenesis [157]. For example, during the progression from NASH to HCC, IL-15 induced FOXO1 (Forkhead Box O1) downregulation and CXCR6 upregulation. This made liver-resident CXCR6^+^ CD8^+^ T cells susceptible to stimulatory factors (including acetate and extracellular ATP). As a result, CD8^+^CXCR6^+^PD1^+^ T cells were induced to attack both parenchymal and nonparenchymal cells in an antigen-independent manner, and this auto-aggressive process further aggravated liver injury and caused a pro-tumor environment. Moreover, CD8^+^CXCR6^+^PD1^+^ T cells have an exhaustive, hyperactivated, and resident phenotype, and ultimately contribute to HCC pathogenesis [113].

Accumulation of LPS itself can also promote HCC by triggering tumorigenesis [97] and T cell-related immunosuppression [158].

Thus, gut dysbiosis disrupts the equilibrium between pro-tumorigenic pathobionts (e.g., *Enterobacteriaceae*) and anti-tumorigenic symbionts (e.g., *Akkermansia*), offering insights into pathogenesis, therapeutic interventions, and biomarker discovery.

## Relationship between gut microbiota and intratumoral microbiota of HCC

In recent years, there have been many studies on the intratumoral microbiota. Intratumoral bacteria are predominantly intracellular, mainly colonizing the cytoplasm of both immune cells and tumor cells [159], and the colonization of intratumoral microbiota was considered due to the hypoxic, immunosuppressed, and nutrient-rich TME [160]. While establishing the relationship between gut and intratumoral microbiota in HCC is complex, emerging evidence suggests a potential correlation.

### Some intratumoral microbiota are derived from gut microbiota

In HCC, the intratumoral microbes mainly originate from the hepatic blood system, biliary system, and normal adjacent tissues [161]. As mentioned above, gut microbiota dysbiosis and gut permeability are aggravated at the onset of HCC [52]. In this situation, gut microbiota has the chance to get into the circulation via Blood vessels in the intestines mucosa and mesentery, and can even translocate into the liver via the portal circulation [49, 61]. In a Lewis lung cancer mouse model [162], gut-derived *A. muciniphila* (which was discussed above, exhibiting several anti-HCC effects) was found migrating into the blood circulation and colonizing local lung cancer tissue. In pancreatic cancer, the gut microbiota is closely related to, and can significantly affect, the intratumoral microbiota [163]. Gut microbes can translocate to and colonize pancreatic tumor tissues, and they control the overall intratumoral microbe composition. They alter immune function, ultimately affecting tumor progression and patient survival [78, 164]. These studies all implied that part of the origin of intratumoral microbiota is the gut microbiota, and the gut microbiota has the potential to mediate tumor progression by influencing the intratumoral microbiota. As the primary organ of HCC, the liver is exposed to the gut microbiota via the portal vein and blood from the gut contributes to nearly 70% of the whole liver blood supply [101, 165]. It is thus natural to consider whether the intratumoral bacteria originated from the gut microbiota and had the potential to influence tumor progression by acting with each other.

### Microbiota profiles in gut and in HCC tumor tissues seem to be consistent

Unlike in the gut microbiota, *Proteobacteria*, *Actinobacteria*, *Bacteroidetes*, and *Firmicutes* were the four dominant bacterial phyla (accounting for up to 90% of the bacteria) in both HCC and matched adjacent nontumor tissues, on the basis of different spatial location and microenvironment [166, 167]. Each type of tumor has a distinct intratumoral microbiome compositive feature [159]. HCC presents characteristic intratumoral microbiota profile that differs from other tumors as well [161, 168].

In a study by Sun et al. involving 28 patients with primary liver cancer (including 11 patients with HCC), *Pseudomonas* was significantly decreased at the genus level (probably due to its antitumor effects) in the tumor tissues compared to the adjacent normal tissues, and *Rhizobiaceae* at the family level and *Agrobacterium* at the genus level were significantly increased in the tumor tissues compared to the adjacent normal tissues [167]. In a study by Huang et al. [161] involving 28 normal liver tissues, 64 peritumoral, and 64 HCC tissues. At the phylum level, the HCC tissues had higher levels of *Proteobacteria*, *Firmicutes*, and *Actinobacteriota*, and lower levels of *Patescibacteria* and *Acidobacteriota* compared to the normal tissues. At the class level, the HCC tissues were enriched in *Gammaproteobacteria*, *Bacilli*, and *Actinobacteria* compared to the normal tissues. In particular, the abundance of *Gammaproteobacteria* in HCC tissues was significantly increased compared to the normal tissues. In the study by He et al. [168] involving 99 HCC and adjacent normal tissues. *Enterobacteriaceae*, *Fusobacterium* and *Neisseria* were significantly increased in the HCC tissues compared to the adjacent normal tissues, *Pseudomonas* was decreased in HCC tissue compared to the norma. Specifically, the proportion of Proteobacteria in HCC was slightly higher and the abundance of *Actinobacteriota* was significantly lower at the phylum level. As for the genus level, the abundances of the genera *Dietzia*, *Faecalibacterium*, *Megamonas*, *Hydrogenophaga*, *Agathobacter*, *Chryseobacterium*, and *Ruminococcaceae* were significantly lower, while the abundances of *Neisseria*, *Clostridia_UCG-014*, *Fusobacterium*, and *Lactobacillus* were significantly higher in the HCC tissues compared to the adjacent normal tissues.

The trends of the same microbiota in gut and in HCC tumor tissues seem to be consistent. In a study by Sun et al. involving 91 HCC patients undergoing hepatectomy, *Actinobacteria*, which was increased in the gut of HCC patients [30] (related to increased stage and worse prognosis [34]), was notably enriched in HCC tissues [166]. At the phylum level, *Proteobacteria* [33], *Firmicutes* [30], Which were significantly increased in the gut of HCC patients, were found to have a higher level in HCC tissue constantly [161]. *Enterobacteriaceae*, the expansion of which in gut was supposed to belong to a distinctive gut dysbiosis profile of HCC [28], was found increased in the HCC tissues, too [168]. At the genus level, *Faecalibacterium* [32], *Agathobacter* [33], and *Ruminococcaceae* [35], which were significantly decreased in the gut of HCC patients, were found to have significantly lower levels in HCC tissue compared to the adjacent normal tissues [168]. *Lactobacillus* was significantly increased in both the gut [41] and the HCC tissues [168] compared to the normal controls.

In summary, though HCC presents characteristic microbiota profiles in both gut and HCC tissues which differ from other tumors, the composition and trend of gut and microbiota had much in common (Fig. 1).

### Gut microbiota and intratumoral microbiota share the same risk-predicters

**Fig. 4:**
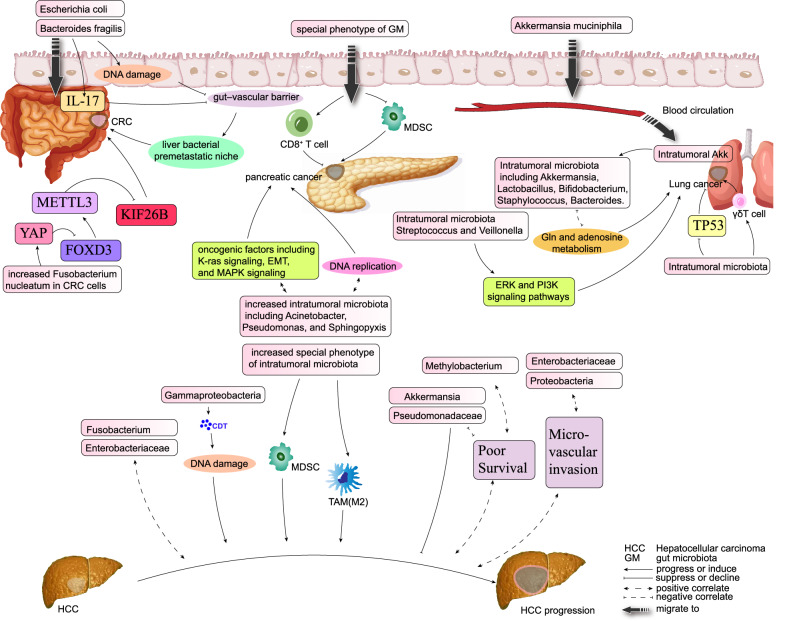
Roles of intratumoral microbiota in HCC and other common tumors. For example, *Gammaproteobacteria* was increased and could secrete cytolethal distending toxin (CDT) and thus cause significant dose-dependent DNA damage which may cause original HCC. Intratumoral microbiota has the potential to intervene in HCC progression by influencing the function of immune cells such as macrophages, T cells and MDSCs. Some downregulated intratumoral microbes might be protective microbes and were significantly positively correlated with some metabolites also decreased in HCC tissues. In lung cancer, gut-derived *Akkermansia muciniphila* migrated into the blood circulation and colonized local lung cancer tissue and then inhibited tumorigenesis mainly by modulating other intratumoral microbiota and activating lung-resident γδ T cells. In CRC, the intratumoral microbiota pks^+^ *Escherichia* and *Bacteroides fragilis* increased metastasis by destroying the gut–vascular barrier by the stimulation of IL-17 and DNA damage. Increased *Fusobacterium nucleatum* in CRC cells activated YAP signaling and downregulated the transcription factor forkhead box D3 (FOXD3), which reduced METTL3 transcription. This subsequently reduced the m^6^A levels of kinesin family member 26B (KIF26B) mRNA and thereby upregulated its expression and inhibited its degradation, which together contributed to CRC metastasis. Improved intratumor microbiota diversity in pancreatic cancer was modulated by the gut microbiota and increased survival due to increased cytotoxic T cells and decreased MDSCs.

Furthermore, there was significant microbial heterogeneity in the intratumoral microbiota between different HCC patients and between multiple tumor foci in the same patient [166]. HCC with different risk factors (with or without HBV, cirrhosis, and so on) or different prognoses also had different intratumoral microbiota [161] (Fig. 4).

For example, *Methylobacterium* in HCC tissue was associated with poor long-term survival in postoperative HCC patients [166]. At either family or genus level in HCC tissue, intratumoral *Pseudomonadaceae* exhibited an anti-tumor effect and was linearly associated with prognosis [167]. A high abundance of *Fusobacterium* in HCC tissue was positively associated with HCC progression [168].

A high level of *Proteobacteria* in the gut is associated with HCC-MVI [44], gut inflammation and dysbiosis [52], basically pathologic status and poor prognosis. Correspondingly, high level of *Proteobacteria* in HCC tissues is associated with the elevation of aspartate aminotransferase (AST), alanine aminotransferase (ALT) and TBA levels, which might also indicate a pathophysiological hepatic condition [166]. Loss of *Akkermansia* in the gut correlates with cellular immunity disorder, liver inflammation and intrahepatic metastasis in HCC, thus poor prognosis [102], lately loss of *Akkermansia* in HCC tissue was found associated with poor long-term survival in postoperative HCC patients as well [166]. A higher abundance of *Enterobacteriaceae* in the gut statistically correlates with HCC-MVI [44], higher HCC stage and worse prognosis [34], higher abundance of *Enterobacteriaceae* in HCC tissue was found related with HCC progression and thus prognosis as well [168]. Although little is known about the crosstalk between the gut microbiota and the intratumoral microbiota, several studies have reported some gut microbiota as HCC risk factors, may play roles in HCC tissue and related to HCC progression and prognosis as well.

### Mechanisms by which the gut microbiota affects the intratumoral microbiota in HCC

The influence of the gut microbiota on the intratumoral microbiome is a multifaceted and intricate process involving various mechanisms.

Firstly, as discussed above, the gut-derived *A. muciniphila* could migrate from the gut and colonize local lung cancer tissue through blood circulation [162], and the gut microbiota can translocate to and colonize pancreatic tumor tissues to control the intratumoral microbiota composition, these facts imply that the gut microbiota has the potential directly colonize the HCC tissue to become and modulate the intratumoral microbiota. Secondly, the gut microbiota contributes to the maintenance of intestinal barrier integrity. Imbalances in the gut microbiota may lead to compromised barrier function, allowing the translocation of bacteria and bacterial products to affect the liver and tumor via the portal venous system [169]. The gut microbiota significantly affects the abundance, distribution and biological role of the intratumoral microbiota by this way. Finally, we have detailed how gut microbiota affects the TME through immune cells [170, 171], metabolites [28, 147], etc., all of which can influence the intratumoral microbiota. The gut microbiota is closely related to the microvascular infiltration [44] and inflammatory microenvironment [45, 49, 61] in tumors, which all play an important role in the growth and reproduction of bacteria in tumor tissues.

## Mechanisms by which the intratumoral microbiota affects HCC progression

Regarding the mechanisms by which the intratumoral microbiota affects HCC progression, although the exact mechanisms are not yet fully known, we summarized three possible ways by which the intratumoral microbiota may affect HCC progression, based on existing studies on HCC and other tumors (Fig. 4).

### Causing DNA damage

The intratumoral microbiota can cause chronic persistent inflammation by disrupting the mucosal barrier via mechanical stimulation, or by secreting toxins that lead to genetic material damage or mutations, upregulating tumor-associated signaling pathways and thus leading to genotoxicity and inducing malignant transformation of cells. For example, HCC tissue had significantly increased *Proteobacteria* at the phylum level and significantly increased *Gammaproteobacteria* at the class level, and the most representative product secreted by *Gammaproteobacteria* is cytolethal distending toxin (CDT) [172], which is a multimeric protein composed of three subunits, CdtA, CdtB, and CdtC. CdtB causes significant dose-dependent DNA damage [173], and may cause original HCC by this way.

### Mediating tumor-related signaling pathways

The abovementioned *Streptococcus* and *Veillonella* were high in HCC and increased with HCC progression [28, 33], and they were also involved in the development of lung cancer related signaling pathways [174]. Intratumoral *Fusobacterium* was increased in HCC [168], and *Fusobacterium nucleatum* in CRC cells activated YAP signaling and downregulated the transcription factor forkhead box D3 (FOXD3), which reduced methyltransferase like protein (METTL) 3 transcription. This subsequently reduced the m^6^A levels of kinesin family member 26B (KIF26B) mRNA and thereby upregulated its expression and inhibited its degradation, which together contributed to CRC metastasis [175]. Although there are no studies on their intratumoral effects in HCC, they (or other functionally similar microbes) have the potential to induce HCC progression by mediating signaling pathways.

### Directly altering the TME

Intratumoral microbes may infiltrate the TME after being stimulated by oxygen and chemotactic gradients [176], The intratumoral microbiota is not only a key component of the TME, but it can also reprogram tumor metabolism to affect tumor invasion and metastasis.

In particular, the intratumoral microbiota affects the immune cells in the TME to influence tumorigenesis and cancer treatment. Intratumor microbes affected the immune checkpoint proteins: in several tumors, fatty acid synthase 2 (fap2) expressed by *Fusobacterium nucleatum* could directly bind to the checkpoint protein TIGIT and thereby inhibit the anti-tumor activity of human NK and T cells [170]. In CRC, enterotoxigenic *Bacteroides fragilis* triggered colon tumorigenesis based on the generation of pro-tumoral MDSCs [177]. In pancreatic tumors, anaerobic bacteria such as *Bacteroides*, *Lactobacillus*, and *Peptoniphilus* may shorten survival time by decreasing the number of tumor-infiltrating T cells [171].

Intratumoral microbiota has the potential to intervene in HCC progression by influencing the function of immune cells such as macrophages, T cells and MDSCs. In 2023, Li et al. [178] analyzed 70 samples, including 29 paired tumor and nontumor tissues from patients with HBV-HCC and 12 liver tissues from patients with chronic hepatitis B (CHB). They established two HCC subtypes based on different intra-tissue microbiota phenotypes and heterogeneity features, namely bacteria- and virus-dominant subtypes, which exhibited distinct clinical manifestations. Patients with bacteria-dominant HCC had larger and more invasive tumors, and worse survival, compared to those with virus-dominant HCC. There was increased M2-type macrophage infiltration in bacteria-dominant HCC compared to virus-dominant tumor HCC. Some risk factors like HBV may result in a unique microenvironment that favors the distinct microbiota colonization, which increases the recruitment and infiltration of CD8 + T cells, monocytic MDSCs (mMDSCs) and polymorphonuclear MDSCs (pmnMDSCs) in HBV-HCC, and finally accelerated the disease progression via inhibiting host antitumor immunity [165].

The close association has been found between different intratumoral microbiota and metabolites in HCC, although the exact mechanism is unclear. In a study analyzing HCC tissues in mice [179], the Kyoto Encyclopedia of Genes and Genomes analysis found that the intratumoral microbial gene’s functions were markedly enriched in the lysosome, glycosaminoglycan degradation, favone and favonol biosynthesis and autophagy yeast than in normal tissues. The Clusters of Orthologous Groups of proteins (COG) functional analysis found that the intratumoral microbial gene’s functions were markedly enriched in glycerol-3-phosphate transport, Fe^3+^-siderophore transport, Sugar (pentulose or hexulose) kinase and Fermentation-respiration switch esterase (FrsA) than in normal tissues. In HCC tissue compared to the normal, the most upregulated metabolites include docosatrienoic acid, FAHFA, L-palmitoylcarnitine, androsterone, citrulline, adrenic acid, myristic acid and so on, the most downregulated metabolites include lactobionic acid, feruloyl putrescine, 6-phosphogluconic acid, S-(methyl) glutathione, thromboxane B1, cholic acid, stercobilin and so on. Furthermore, in the analysis investigating the association between the top 20 diferrential metabolites and the top 10 diferrential intratumoral bacteria, top abundant intratumoral bacteria *Allobacillus sp SKP4 8* and *Ralstonia sp UNC404CL21Col* showed significant positive correlations with most metabolites, including citrulline, cytidine 5′-monophosphate (hydrate), indole-3-lactic acid, 2′-O-methylguanosine, cytidine5′-monophosphate, L-(+)-Citrulline, cis-4-HydroxyD-proline, and myristic Acid, while showed significant negative correlations with a-Lactose and N-acetyl-Dglucosamine. However, the top abundant normal-tissue bacteria *Pseudomonas koreensis* and *Pseudomonas psychrotolerans* were in negative correlation with most metabolites, and the trend was opposite to the *Allobacillus sp SKP4 8* and *Ralstonia sp UNC404CL21Col*. Some downregulated intratumoral microbes might be protective microbes and were significantly positively correlated with some metabolites also decreased in HCC tissues, and they may be involved in the synthetic and metabolic processes of some protective compound metabolites [180].

### Modulating HCC metastasis

The intratumoral microbiota plays an important role in metastasis. Intravenous antibiotics selectively inhibit the intratumoral (not gut) bacteria, while oral antibiotics deplete both types of bacteria. In a mouse breast cancer model, intravenous antibiotics decreased metastasis while not affecting the primary lesion, while oral antibiotics decreased both metastasis and primary lesion formation, hinting at the key role of the intratumoral microbiota in metastasis [181]. In HCC, the situation is more complicated. The liver is exposed to gut microbiota metabolites and products carried by the blood, as 70% of the whole liver blood supply is from the gut [101]. In many mouse models, oral antibiotics had liver-selective antitumor effects by restraining the gut microbiota that specifically influenced intrahepatic tumors. These mouse models included the spontaneous HCC transgenic model, the subcutaneous implantation model (liver metastases were reduced but not the primary foci), and the intrasplenic tumor injection model (liver metastases were reduced, while lung metastases were increased by tumor cell tail vein injection in a different model). The liver-selective antitumor effects may partly be the result of the gut microbiota using bile acids to control NKT cell infiltration in the liver [101].

Additionally, tumor-derived exosomes can transfer miRNAs and proteins and promote tumor metastasis through multiple mechanisms, such as remodeling TME, promoting EMT and inhibiting the antitumor immune response [182–184]. Tumor cells infected by bacteria may secrete more exosomes, thus accelerating the metastasis of the tumor [185, 186].

There may also be a synergistic effect of gut flora and intratumor flora in promoting tumor metastasis, because bacteria of the same genus tend to act accordingly in tumor metastatic progress whether in the gut or in tumor cells. *Fusobacterium nucleatum* in gut inhibited the anti-tumor activity of human NK cells and T cells [170], in tumour cells it increased the mobility of these cells mobility, rendered them more resistant to stress in the circulation, and finally promoted tumour metastasis [187].

### Other anti-tumor effects

Intratumoral microbiota could protect tumor cells from fluid shear stress during cell migration [188]. In the research of intratumoral microbiota in a murine spontaneous breast cancer model, intratumor bacteria colonized in circulating tumor cells were found to defending the fluid shear stress by reorganizing actin cytoskeleton resulted in metastasis process [181]. *S*. *xylosus*, *L*. *animalis*, and *S*. *cuniculi* could increase the survival of breast cancer cells in the lung from 3.4 to 6.4 fold [181], none of them were reported in HCC to take a significant role in tumor cells. This field is still relatively new and needs to be explored how specific bacteria affect what specific tumour type metastates to specific organs.

It is clear that intratumoral microbiota are not exclusively pro-tumor, they can activate a bacterial antigen-specific response, which can be used not only to amplify the immune response to tumor antigens, thus increase the killing power of the tumor [188]. Briefly, bacteria could destroy tumor cells after targeting and localizing them and then release tumor-associated antigen and damage-associated molecular pattern to recruit or activate immune cells [189]. For example, in melanoma, researchers found that some microbial antigens were homologous to tumor antigens and thus they share similar antigenic epitopes [190]. Subsequently, the tail length tape measure protein of the bacteriophage *Enterococcus hirae*, and SVYRYYGL (SVY) epitope on the *Bifidobacterium breve* was similar to the tumor antigen and might cross-react, promoting T-cell responses [191]. It is of great potential in tumor therapy if we improve the tumor microenvironment by inhibiting pro-tumor intratumoral microbiota and stimulating tumor immune response by increasing some special intratumoral bacterial epitopes.

In summary, this review discussed the role of gut microbiota in the HCC progression, the relationship between gut microbiota and intratumoral microbiota and their interaction in HCC. Both the gut and intratumoral microbiota have a two-sided role in HCC progression, and it is of great potential in tumor therapy if we inhibit pro-tumor microbiota and stimulate tumor immune response by increasing anti-tumor microbiota.

## Discussion

The mechanisms by which gut dysbiosis leads to the progression and metastasis of many tumors, including HCC, have been studied for many years. The liver absorbs nutrients from the portal vein, and the liver also receives metabolites from the gut microbiota via the gut–liver axis [132]. The gut–liver axis is the main pathway through which the gut microbiota influences the development of liver disease [132]. The main components of this axis are (1) a mucus layer containing commensal microorganisms, secreted IgA, and antimicrobial peptides; (2) a layer of intestinal epithelial cells with tight junctions between neighboring cells; and (3) a lamina propria with a resident population of innate and adaptive immune cells [192]. The gut microbiota influences HCC mainly by modulating bile acid metabolism, affecting nutrient metabolism, and altering levels of metabolic products.

Importantly, most of the gut microbiota are nonharmful. In a balanced state or under external conditions, the gut microbiota, and various biomolecules in the internal environment often interact with each other and exert synergistic anti-tumor effects. For example, PPARs are a family of transcription factors that govern essential metabolic activities. One of the members, PPARδ, plays a critical role in the antitumor effect of butyrate and berberine. Berberine increased BAX, cleaved caspase 3, and decreased BCL2 expression to suppress HCC development dependent on PPARδ. On the other hand, berberine activated the PPARδ transcriptional function to facilitate the binding of PPARδ to the promoters of apoptotic genes such as caspase 3, BAX, and BCL2. Moreover, berberine restored the dysregulated gut microbiota induced by the liver tumor burden, rescuing the level of the gut microbial metabolite butyric acid. Butyric acid in return enhanced the efficacy of berberine by reducing PPARδ degradation via inhibiting the ubiquitin–proteasome system [153]. As the important link in the middle, the balance of the gut microbiota is crucial to disease progression, and disruption of the balanced gut microbiota can strongly destroy the overall anti-tumor effect, and even produce a tumor-promoting microenvironment.

Metabolites, such as SCFAs, are double-edged in HCC as well. For example, in NAFLD-HCC, the gut microbiota induced high levels of SCFAs such as butyrate, which was correlated with increased IL-10^+^ Tregs but decreased CD8^+^ cells [28], seemingly not consistent with its anti-HCC effect. In another example, acetate induced by various factors exerted different effects on HCC, which has been mentioned above [148, 149]. The paradox of the tumor-promoting or tumor-suppressing functions of SCFAs suggests that to accurately elucidate the functions of microbial metabolites, it is important not to lose sight of the environments in which the metabolites function, as well as the different types and concentrations of the metabolites. For example, when concentrations of SCFAs are within their normal physiologic ranges, they significantly inhibit the development of colon cancer. Increasing the dose of SCFAs beyond the tolerance threshold promotes tumor progression, and the same mechanism and principle may exist in HCC, waiting to be explored and investigated [193, 194].

Although little is known about the crosstalk between the gut microbiota and the intratumoral microbiota, several studies have reported on the biological functions of the gut microbiota, via effects on the intratumoral microbiota, in various cancers.

In a Lewis lung cancer mouse model [162], gut-derived *A. muciniphila* (which was discussed above, exhibiting several anti-HCC effects) migrated into the blood circulation and colonized local lung cancer tissue. *A. muciniphila* then inhibited tumorigenesis partly by modulating the tumoral symbiotic microbiota. A higher level of gut *A. muciniphila* (due to gavage) increased the abundance of intratumoral *Akkermansia*, *Lactobacillus*, *Bifidobacterium*, *Staphylococcus*, and *Bacteroides*, and these symbiotic bacteria likely played an inhibitory role in glutamine (Gln) and adenosine metabolism, thus helping to block local tumor progression in the lungs. Alterations in the lung intratumoral microbiota can influence lung cancer progression via many routes. The lung cancer microbiota may activate lung-resident γδ T cells to increase inflammation associated with lung adenocarcinoma. In detail, the microbes may stimulate MyD88-dependent myeloid cells to produce IL-1β and IL-23, activating Vγ6^+^ Vδ1^+^ γδT cells to produce factors such as IL-17, leading to inflammation and tumor cell proliferation [195]. Some intratumoral microbes in squamous cell carcinoma caused epithelial TP53 mutations [196]. Thus, the gut microbiota significantly alters the intratumoral microbiota in lung cancer, and the intratumoral microbiota influences lung cancer progression Through a variety of means including glutamine (Gln) and adenosine metabolism, γδ T cells, interleukin and TP53. Furthermore, the roles of the intratumoral microbiota are more complicated than “pro-tumor” v.s. “anti-tumor”. In the Lewis lung cancer mouse model, *Staphylococcus* and *Lactobacillus*, which may block local tumor progression at the primary tumor site [162], were reported to colonize tumor cells that migrated from the primary site to metastatic foci and thereby significantly increased the number of metastatic foci in a spontaneous breast tumor model, and they did not influence the primary site [181].

As for colorectal cancer (CRC), there were significant differences in the intratumoral microbiota between tissues with and without KRAS mutation or microsatellite instability (two key factors underlying CRC progression and prognosis). This indicates the close associations of intratumoral microbial heterogeneity with genetic alteration and CRC development [197]. When CRC cells began to metastasize, the intratumoral microbiota increased metastasis by destroying the gut–vascular barrier and building a bacterial premetastatic niche in the liver [198]. The mechanism involves increased genotoxic pks^+^ *E. coli*-encoded colibactin-synthesizing enzyme levels that promote CRC metastasis [199]. This may be driven by *Bacteroides fragilis*, as colibactin lead to increased IL-17 and DNA damage in colonic tissue and worsens prognosis [200]. *E. coli* in CRC disrupted the gut–vascular barrier reached the liver via the blood and then facilitated liver metastasis [201].

Improved intratumor microbiota diversity in pancreatic cancer was modulated by the gut microbiota and increased survival due to increased cytotoxic T cells and decreased MDSCs [163]. Increased *Pseudoxanthomonas*, *Streptomyces*, *Saccharopolyspora*, and *Bacillus clausii* in pancreatic tumor tissue predicted better survival [163], while anaerobic bacteria (such as *Bacteroides*, *Lactobacillus*, and *Peptoniphilus*) were significantly positively associated with shorter survival time [171]. Regarding the potential mechanism in pancreatic cancer, increases in the genera *Acinetobacter*, *Pseudomonas*, and *Sphingopyxis*, were positively correlated with DNA replication and oncogenic factors including K-ras signaling, EMT, and MAPK signaling, while being negatively correlated with bile acid metabolism, pancreatic beta cells, and pancreatic secretion [202].

These studies may provide us with ideas to explore how gut microbiota interacts with intratumor microbiota and together affects tumor progression. This can help us understand more deeply the correlation between intratumoral microbiota and gut microbiota, as well as help us to further explore the possible mechanisms of the intratumoral microbiota modulating other types of tumors like HCC. Many of the possible mechanisms that how intratumoral microbiota improve HCC progression that we summarized in the previous section are based on these studies.

Intratumoral microbiota also has two sides on tumors, some of the intratumoral microbiota can inhibit tumor progression as well. Some microbial antigens were homologous to tumor antigens [190], thus can stimulate anti-tumor immune reactions [191]. It is of great potential in tumor therapy if we improve the tumor microenvironment by inhibiting pro-tumor intratumoral microbiota and stimulating tumor immune response by increasing some special intratumoral bacterial epitopes.

Both the gut and intratumoral microbiota have a two-sided role in HCC progression, fully understanding and mastering the complex biological roles of these microbiota will provide a theoretical foundation for the potential clinical application of gut and intratumoral microbiota in the treatment of HCC.

The gut microbiota plays a significant role not only in the initiation and progression of HCC but also exerts a substantial influence on its treatment through these biological mechanisms. For example, the treatment options for HCC patients including liver surgical resection, percutaneous ablation, liver transplantation, radiation, as well as trans-arterial and systemic therapies, the clinical decision making based on the patient’s tumor stage, liver function, and performance status. Above these therapies, nowadays trials aim to address the efficacy of immunotherapy plus targeted therapy or not as adjuvant or neo-adjuvant treatment, with initial promising results from phase 2 trials [203–205] and phase 3 trials [206]. To the advanced unresectable HCC, immunotherapy and targeted therapy are supposed to be the main treatment [207, 208]. The role of gut microbiota and its metabolites in modulating local and systemic immune responses, thereby impacting the cancer-immune axis and influencing the efficacy of immunotherapy for HCC, is emerging as a topic of significant interest in the field [209, 210]. Based on these facts, the significant therapeutic role of the gut microbiota in HCC warrants further in-depth investigation and exploration.
